# Fair and unfair punishers coexist in the Ultimatum Game

**DOI:** 10.1038/srep06025

**Published:** 2014-08-12

**Authors:** Pablo Brañas-Garza, Antonio M. Espín, Filippos Exadaktylos, Benedikt Herrmann

**Affiliations:** 1 Business School, Middlesex University London, London NW4 4BT, UK; 2 GLOBE, Departamento de Teoría e Historia Económica, Universidad de Granada, Campus de la Cartuja s/n, 18071 Granada, Spain; 3 Istanbul Bilgi University, BELIS, Murat Sertel Center for Advanced Economic Studies, 34060 Eyup Istanbul, Turkey; 4 School of Economics, University of Nottingham, University Park Nottingham, Nottingham, NG7 2RD, UK

## Abstract

In the Ultimatum Game, a proposer suggests how to split a sum of money with a responder. If the responder rejects the proposal, both players get nothing. Rejection of unfair offers is regarded as a form of punishment implemented by fair-minded individuals, who are willing to impose the cooperation norm at a personal cost. However, recent research using other experimental frameworks has observed non-negligible levels of antisocial punishment by competitive, spiteful individuals, which can eventually undermine cooperation. Using two large-scale experiments, this note explores the nature of Ultimatum Game punishers by analyzing their behavior in a Dictator Game. In both studies, the coexistence of two entirely different sub-populations is confirmed: prosocial punishers on the one hand, who behave fairly as dictators, and spiteful (antisocial) punishers on the other, who are totally unfair. The finding has important implications regarding the evolution of cooperation and the behavioral underpinnings of stable social systems.

A wealth of interdisciplinary research has attested to the pivotal role of fairness norms in explaining cooperation amongst unrelated individuals^1^,^2^,^3^,^4^,^5^,^6^. The Ultimatum Game (UG)^7^ has been one of the most prolific set-ups for unraveling the nature of human fairness over the last years^1^,^3^,^5^,^8^,^9^,^10^,^11^,^12^,^13^,^14^,^15^,^16^,^17^,^18^. In this game, one player (the proposer) proposes a way to split a sum of money with another player (the responder). If the responder accepts the offer, both players are paid accordingly; if she rejects the offer, neither player is paid. The rejection of positive, albeit low offers is considered to be an expression of costly punishment of unfair behavior able to enforce the social norm of fairness^2^,^3^. Thus, the prevailing view is that individuals' (prosocial) preferences for fairness motivate the rejection of low offers in the UG. Such an argument has been used to support the strong reciprocity model of the evolution of human cooperation^4^,^19^,^20^,^21^. However, recent evidence now challenges this interpretation^22^.

Indeed, less prosocial motivations may be behind UG rejections. Rejections are equally compatible with competitive, spiteful motives^23^,^24^,^25^,^26^. A spiteful responder who is concerned with her own relative standing prefers a zero-zero outcome over one that leaves her below the proposer. Thus, she will reject any offer below the equal split, just like an individual concerned with the fairness norm. This implies that the mere observation of UG behavior is insufficient to determine the motivation of rejections. A crucial factor that should not be overlooked points directly to the punisher^27^,^28^: does she herself comply with the norm?

Recent research on cooperation games reveals that costly punishment is not only used by cooperators but also by non-cooperators who punish (other) non-cooperators^29^, cooperators^30^,^31^ or both^28^. These punishment patterns, which cannot be reconciled with fairness motives, have been traced back to competitive, spiteful individuals aiming to increase their own relative payoff. Such findings are particularly important insofar as theoretical and empirical evidence demonstrates that the commons can be destroyed by the presence of “spiteful (unfair) punishers”, which can ultimately turn a sanctioning institution into a detrimental force for public cooperation (30,31,32,33,34; see^35^ for a recent overview).

This note investigates experimentally to what extent fair and unfair punishers coexist in the UG. To disentangle “prosocial” and “antisocial” types of punishment, this paper combines the rejection behavior of subjects in the UG with their decisions as dictators in a Dictator Game (DG)^36^. The DG is identical to the UG except that the second player is now passive, that is, she cannot reject the offer. As a result, generous offers by dictators are genuinely prosocial. A prosocial individual concerned about fairness will split the pie equally in the DG *and* reject unequal offers in the UG. A spite-driven, antisocial individual, however, will still reject unequal offers but transfer nothing in the DG (hence being totally unfair) in order to achieve the highest payoff differential. Note that pure selfishness also predicts a zero-transfer in the DG but never the rejection of any positive offers in the UG. Therefore, the rejection of unequal but positive offers combined with zero-transfers in the DG is an unequivocal symptom of competitive spite.

We report data from two large-scale experimental studies. Study 1 (n = 754) is a survey-experiment employing a representative sample of a city's adult population which was carried out at the participants' households. The pie to be split was €20 in each game. For UG responses, the strategy method was used in which the responder states whether she accepts/rejects any possible offer beforehand. Study 2 (n = 623) is a replication of Study 1 in the laboratory employing university students (freshmen) as subjects (see Methods).

## Results

**Figure 1:**
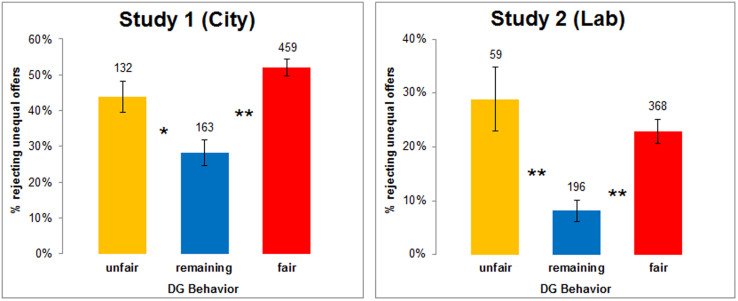
Willingness to reject unequal offers by DG groups. Left [right] panel for Study 1 [2]. The horizontal axis depicts behavior in the DG: unfair (offer 0%), remaining (offer between 0 and 50%), fair (offer 50%). The numbers on top of the bars denote the total number of observations in each group. The vertical axis represents the percentage of individuals (± SE) who reject offers below 50% in the UG, i.e. whose minimum acceptable offer is the equal split (mean percentage: 45.49% in Study 1, 18.78% in Study 2). * *p* = 0.005, ** *p* < 0.001.

Figure 1 breaks down the sample into three groups according to participants' decisions in the DG: “unfair” refers to participants who offer zero in the DG, “fair” refers to those who make an equal split, while those who make an offer in between the two are labeled “remaining”. For each group, the figure displays the percentage of responders who reject offers below the equal split in the UG. Study 1 [2] is captured by the left [right] panel.

The data clearly demonstrate that it is not only the “fair” but also the (totally) “unfair” dictators who reject unequal offers significantly more often than the “remaining” group (Probit model controlling for order effects in decisions; fair vs. remaining: *p* < 0.001 in Study 1 and 2; unfair vs. remaining: *p* = 0.005 in Study 1, *p* < 0.001 in Study 2; see model 1 in Table S1 for Study 1 and Table S2 for Study 2 in the Supplementary Information (SI)). What is more, both groups are similarly likely to reject an unequal offer (*p* = 0.123 in Study 1, *p* = 0.356 in Study 2). As analyzed in more detail in the SI (see Figure S3), there is a statistically significant U-shaped, non-linear relationship between the two variables in both samples (all *p*s < 0.001) when using the offers in the DG as a continuous explanatory variable (rather than comparing between the three DG groups). Furthermore, having decided first as dictator or as responder does not affect the reported relationship (no significant main or interaction order effects are observed in any study: all *p*s > 0.16; see SI).

Thus, fair and unfair punishers coexist in the UG. In addition, in both samples fair dictators are more numerous than unfair ones (see the numbers on the top of the bars in Figure 1; the percentage of fair dictators is significantly higher than the percentage of unfair dictators according to a two-tailed binomial test: *p* < 0.001 in both studies). This implies that fairness-based punishment is more frequent in both samples – which, nevertheless, should not necessarily be the case in samples taken from other populations/societies^30^ (also, as discussed in the SI, methodological factors might influence these proportions). Indeed, among the UG responders who reject unequal offers in Study 1 [2], 17% [15%] are unfair dictators while 70% [72%] are fair dictators (these percentages are also significantly different according to a two-tailed binomial test: *p* < 0.001 in both studies). Importantly, note that the relationship between DG offers and UG rejections holds even in the presence of differences between the two samples. In particular, in Study 1 the proportion of unfair dictators as well as the likelihood of rejecting unequal UG offers is higher compared to Study 2 (in both cases, two-tailed Fisher's exact test yields *p* < 0.001).

## Discussion

The results show that punishment decisions in the UG are indistinguishably “prosocial” and “antisocial”; it is not one or the other, but both kinds of human behavior that shape the outcomes of the UG. Such a finding has important implications in interpreting previous results and designing future research.

One prominent example lies in the realm of behavioral and social neuroscience, where data from rejections in the UG have been extensively used to investigate the neurobiological basis of costly punishment. This research has implicated the brain areas responsible both for negative emotional processes (e.g., the anterior insula) and for executive control (e.g., the dorsolateral prefrontal cortex) in rejection behavior. Yet, there is much debate on the exact role of executive control. Some studies appear to indicate that executive control must be exerted to override the emotional impulse to punish unfairness at personal cost^5^,^37^ whereas others suggest that it is the selfish impulse to accept an unfair offer which must be overridden in order to impose fairness through rejection, thus implying that punishment is an act of self-control^8^,^38^. Recently, more studies have shed light on these apparently contradictory observations^13^,^14^,^15^,^16^ but the debate is far from closed. The results presented in this note indicate that there is a non-negligible fraction of rejections that are rooted not in normative, fairness-based judgments but instead in competitive, spiteful desires. It would in fact be hard to claim that a common neural mechanism underlies these extremely different natures of rejection behavior. Instead, one of the two might be overrepresented in some databases – which might have been due to the small sample sizes typically featured in brain studies – and, as suggested in^29^, this could explain part of the above controversy. Note also that the proportion of prosocial and antisocial punishers may vary dramatically across societies^30^,^35^,^39^.

Thus, in order to unravel the neurobiological basis of costly punishment, researchers should carefully investigate not only which behavior gets punished but also *who is* the punishing individual. However, this cannot be addressed using the standard UG as the only information researchers obtain from responders is whether they accept or reject a given proposal (or a number of them). Other experimental settings, or the combination of UG rejections with subjects' behavior in other frameworks, should be employed.

Additionally, the results are also important from the viewpoint of evolutionary biology and the social sciences. Costly punishment has been shown to be crucial in promoting cooperation^40^,^41^,^42^,^43^. Nevertheless, in the presence of spiteful punishers, social efficiency becomes difficult to sustain since spiteful behavior often leads to escalating conflict rather than to lasting cooperation. When sanctions are not used as norm-enforcement devices but instead at the service of dominance- or conflict-seeking behavior, their effects over social stability can be perverse^30^,^31^,^32^,^44^. If the punisher lacks the legitimacy to teach a moral lesson – because she does not comply with the social norm herself – the punished individual can view punishment as unjustified coercion. This might activate the mechanisms involved in competition with conspecifics instead of those involved in norm compliance, thus paving the way to inefficient, corrupt societies^45^,^46^ rather than to efficient, cooperative ones^47^. In fact, corruption among the responsible for the enforcement of rules is recognized as a major source for the failure of social institutions^48^.

Therefore, special care has to be taken in the interpretation of rejection behavior as a mechanism to enforce the norms implicated in the maintenance of stable social systems. Extending the argument to the field of institutional design, failing to recognize the possible duality of motives behind punishment behavior in bilateral bargaining interactions can lead to less-than-optimal, or even counter-effective incentive mechanisms.

## Methods

The details of the survey-experiment have been reported elsewhere^49^. In both studies, subjects made their decisions in the UG (both roles) and the DG in random order. Subjects' decisions as proposers in the UG are not being used here as a measure of fairness since generous offers might equally be motivated by strategic self-interest (avoidance of rejection) and by other-regarding concerns, thus making them difficult to interpret^50^ (indeed, zero offers in the UG are extremely rare). In contrast, the interpretation of subjects' offers in the DG is straightforward because they are not influenced by strategic concerns.

In the DG, subjects had to split a pie of €20 between themselves and another anonymous participant. Subjects decided which share of the €20 (in €2 increments) they wanted to transfer to the other subject. For the role of responder in the UG the strategy method was used^51^. That is, subjects had to state their willingness to accept or reject each of the following proposals (proposer's payoff [€], responder's payoff [€]): (20, 0); (18, 2); (16, 4); (14, 6); (12, 8); (10, 10). After making their decisions, participants in each study were randomly matched and one of every ten was selected for real payment (see Supplementary Information).

For the statistical analyses, we use Probit regressions with the likelihood that a subject rejects any unequal offer (i.e. whether her minimum acceptable offer is the equal split) in the UG as the dependent variable and DG behavior as the explanatory variable. Using the same database, a similar approach was employed in Staffiero et al.^52^ for the study of the motivational drives behind the acceptance of zero offers in the UG.

All participants in the experiments reported in the manuscript were informed about the content of the experiment prior to participating. Verbal informed consent was obtained from participants in the city experiment (Study 1) since literacy was not a requirement to participate (this was necessary to obtain a representative sample), and all the instructions were read aloud by the interviewers. Written informed consent was obtained from participants in the lab experiment (Study 2). Anonymity was always preserved (in agreement with Spanish Law 15/1999 on Personal Data Protection) by randomly assigning a numerical code to identify the participants in the system. No association was ever made between their real names/addresses and the results. As is standard in socio-economic experiments, no ethic concerns are involved other than preserving the anonymity of participants. This procedure was checked and approved by the Vice-dean of Research of the School of Economics of the University of Granada; the institution hosting the experiments.

## Author Contributions

P.B.G., A.M.E., F.E. and B.H. contributed equally to all parts of the research.

## Supplementary Material

Supplementary Information Supplementary Information
