# Supplementary material for: Fair and unfair punishers coexist in the Ultimatum Game
Source: Sci Rep. 2014 Aug 12;4:6025. doi: 10.1038/srep06025 (PMC4129421; doi:10.1038/srep06025)
Supplement: Supplementary Information [file srep06025-s1.pdf]

Supplementary Information (SI) for

Fair and unfair punishers coexist in the Ultimatum Game

Pablo Brañas-Garza, Antonio M. Espín, Filippos Exadaktylos, Benedikt Herrmann

S1. Experimental procedures

After completing a questionnaire (identical across studies) to collect socio-economic and psychological information, subjects made decisions in a Dictator Game, an Ultimatum Game (both roles) and a Trust Game (both roles).[*] Since every subject made all five experimental decisions, the order both between and within games was randomized across subjects, resulting in 24 different orders (the two decisions of the same game were always set aside). In each study, one out of ten subjects was randomly selected to be paid for one randomly chosen game and role. Matching and payment took place within the next two weeks and the average earnings among winners were €9.60 in Study 1 and €10.43 in Study 2. In both cases the max. payoff was €40.

Study 1 (City) took place from November 23rd to December 15th 2010 in Granada, Spain. A total of 835 individuals between 16 and 91 years old participated (average age 38.5±17.5 [SD], 53.9% female, 22% college students). The experiment was carried out in the subjects' houses and data were collected by 108 pairs of senior-year university students who had previously undergone a 10-hour training program on the methodology of field experiments. The sample was obtained via a stratified random method, which ensured its representativeness. Extended information on the sampling procedures and the resulting sample has been previously reported (S1) and is also available at https://sites.google.com/site/experimentalcity/home.

[*] An English translation of the questionnaire and the complete experimental instructions (Study 1) are available at https://sites.google.com/site/experimentalcity/home.

The experiment in Study 2 (Lab) took place during October 2011 in the Granada Lab of Experimental Economics EGEO at the University of Granada. In this study, all first-year students at the School of Economics were invited to participate in the experiment during their first week of problem-solving classes. Thus, participants in Study 2 were "pseudo-volunteers".[†] A sample of 659 students participated (average age 19.1±2.3 [SD], 58.1% female) in a total of 27 sessions.

In the Dictator Game (DG), subjects had to split a pie of €20 between themselves and another anonymous participant (S3). Subjects decided which share of the €20 (in €2 increments) they wanted to transfer to the other subject. For the role of responder in the Ultimatum Game (UG), the strategy method was employed (S4). That is, subjects had to state their willingness to accept or reject each of the following proposals (proposer's payoff [€], responder's payoff [€]): (20, 0); (18, 2); (16, 4); (14, 6); (12, 8); (10, 10).

With this method we obtained the minimum acceptable offer (MAO) of each subject. For the analyses in this paper, only subjects with complete and reliable observations in both decisions are considered. This involves excluding from the sample those subjects with missing values in any of the two decisions and those making inconsistent choices (i.e., those following non-monotonic patterns or making multiple switching between acceptance and rejection) as responders in the UG. Finally, we excluded subjects who transferred more than 50% of the pie in the DG (5.3% in Study 1 and 3.4% in Study 2) since our interest is in fairness-based versus spite-based behavior; transfers above the equal split, although obviously generous, cannot be easily linked to any fairness norm. The resulting sample sizes used in the analyses are n=754 in Study 1 and n=623 in Study 2.

An English translation of the experimental instructions for Study 1 (only minor adjustments were made for Study 2) can be found in section S3. In Study 1, the instructions were read aloud by the interviewer and subjects had to write their decisions on a decision sheet which was subsequently placed in a sealed envelope (see Figure S1 for an English

[†] It has been found that pseudo-volunteer students are more generous than the typical student subjects who volunteer in laboratory economic experiments (S2). Indeed, in the present sample DG offers are above what is normally observed within student samples.

translation of the decision sheet). For Study 2, the procedures were computerized using an online web-based system.

Figure S1. Decision sheet (Study 1)

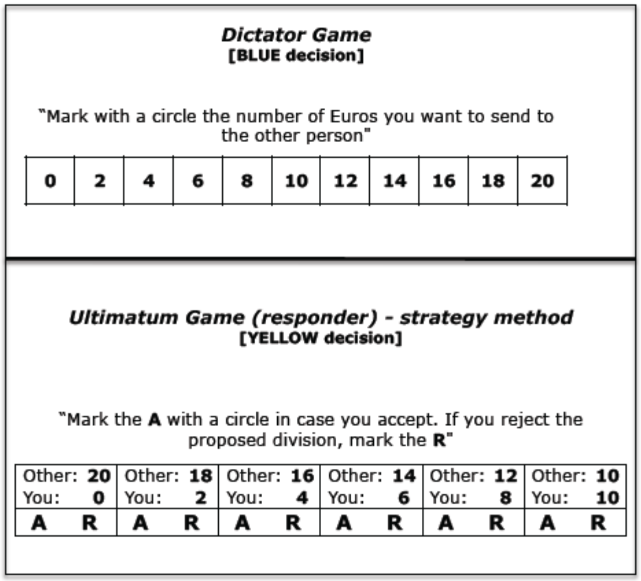
Dictator Game
[BLUE decision]

"Mark with a circle the number of Euros you want to send to the other person"

**0**	**2**	**4**	**6**	**8**	**10**	**12**	**14**	**16**	**18**	**20**

Ultimatum Game (responder) - strategy method
[YELLOW decision]

"Mark the **A** with a circle in case you accept. If you reject the proposed division, mark the **R**"

Other: **20** You: **0**		Other: **18** You: **2**		Other: **16** You: **4**		Other: **14** You: **6**		Other: **12** You: **8**		Other: **10** You: **10**	
**A**	**R**	**A**	**R**	**A**	**R**	**A**	**R**	**A**	**R**	**A**	**R**

S2. Supporting analyses

The average (±SD) DG offer and UG MAO were, respectively, €7.40±3.87 and €7.07±3.53 in Study 1, and €7.99±3.18 and €6.10±3.04 in Study 2. This average behavior is consistent with previous findings (S5, S6). At the same time, in both studies the number of zero offers in the DG was relatively small compared to the standard figures. This might be driven by a cultural factor, but it can also further be fostered by the particular methodology employed in the study, i.e. by the fact that it was a survey-experiment containing several tasks and that the subjects were pseudo-volunteers. Even if this possibility cannot be ruled out, there is no reason to suggest that such an effect might have also influenced the relationship between the behavior as dictator in the DG and as responder in the UG.

Figure S2 presents the histograms of DG (panels a and b) and MAO (panels c and d) decisions for the final sample of both studies. It can be observed that the distributions of choices in the DG were similar in the two studies although €0 transfers were more frequent in Study 1, while €8 (40% of the pie) transfers were more frequent in Study 2.

On the other hand, the distribution of MAOs is clearly different between the studies, particularly in terms of the subjects' willingness to reject unequal offers, which constitutes the focus of the paper: while 45.49% of subjects in Study 1 set their MAO to €10 (i.e., rejecting any offer below the equal split), the percentage sharply decreases to 18.78% in Study 2. Students in Study 2 appear to be "less strict" responders, setting their MAOs to €6 or €8 more frequently than subjects in Study 1. These differences, however, do not discredit the basic results. Rather the opposite: as it is discussed later, despite using two samples with different rejection behavior, the *relationship* between the subjects' willingness to reject unequal offers in the UG and their behavior in the DG follows an almost identical pattern in the two studies.

Figure S2. Distribution of subjects' choices in the games

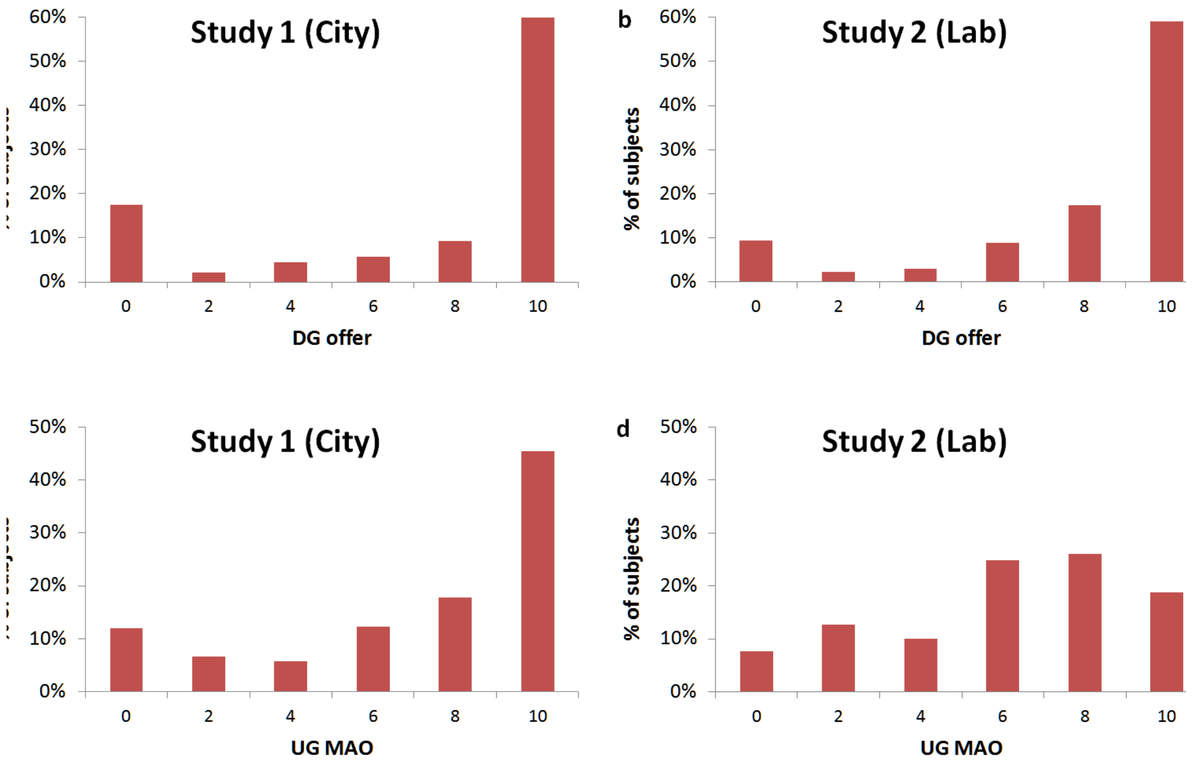

Nevertheless, in terms of rejecting very low offers, the differences between Study 1 and Study 2 are less striking. Offers of €4 or less were rejected by 75.6% and 69.7% of

subjects, respectively. This is also congruent with previous literature as rejection rates up to 80% have been observed for offers below 25% of the stake (S5).

Tables S1 (Study 1) and S2 (Study 2) present the estimations of the models used to support the results of the paper. In all regressions, the dependent variable is the likelihood of rejecting an unequal offer in the UG, that is, whether the subject set her MAO to €10. The tables present Probit estimates for the comparison between "unfair" (DG transfer=0) and "remaining" (0<transfer<10) dictators, between "fair" (transfer=10) and "remaining" dictators, and between "unfair" and "fair" dictators. The coefficients together with their standard errors and *p*-values are displayed for each comparison. For the two first comparisons (unfair vs. remaining and fair vs. remaining) we introduced dummies for unfair and fair, whereas the comparison group is remaining. For the last comparison, we introduced dummies for unfair and remaining, with fair as the comparison group, and report the coefficient of unfair.

Column (1) of both tables shows regressions controlling for the order in which decisions were made. These are the results reported in the main text. Column (2) presents estimates for regressions without controlling for order effects. In columns (3) and (4) the same regressions are repeated by adding a set of controls consisting of basic socio-demographic variables (age, gender, educational level, and household income) and a proxy for cognitive abilities (number of correct responses to a series of simple mathematical questions). The samples are slightly reduced for the regressions displayed in columns (3) and (4) due to missing values in control variables. As can be observed, the results are robust to all of these specifications in both studies. The likelihood of "fair" and "unfair" dictators rejecting unequal offers in the UG is not significantly different, while both groups are significantly more likely to reject unequal offers than the "remaining" dictators. We also checked the effect of clustering standard errors by interviewer in Study 1, as in (S1). *P*-values slightly increase in all cases but none of the significant comparisons exceeds p=0.013. Two-tailed Fisher's exact tests also yield nearly identical results (departures from the *p*-values shown in column (1): unfair vs. remaining, p=0.007; unfair vs. fair, p=0.114 in Study 1; unfair vs. fair p=0.324 in Study 2).

Table S1. Impact of DG behavior on the likelihood of rejecting unequal offers (Study 1)

	(1)	(2)	(3)	(4)
unfair – remaining	0.446*	0.424*	0.481*	0.457*
	(0.157)	(0.151)	(0.163)	(0.156)
p-value	0.005	0.005	0.003	0.003
fair – remaining	0.647**	0.628**	0.631**	0.610**
	(0.124)	(0.120)	(0.129)	(0.124)
p-value	0.000	0.000	0.000	0.000
unfair – fair	-0.201	-0.204	-0.150	-0.153
	(0.131)	(0.124)	(0.136)	(0.129)
p-value	0.123	0.100	0.271	0.234
order effects	YES	NO	YES	NO
pseudo R^2	0.0565	0.0275	0.0705	0.0399
log likelihood	-490.208	-505.274	-470.687	-486.208
LR (chi^2)	58.71**	28.58**	71.44**	40.39**
Controls	no	no	yes	yes
observations	754	754	735	735

Notes: Probit estimates. Standard errors in brackets. *, ** indicate significance at the 0.01 and 0.001 levels, respectively. In columns (3) and (4), controls are: age, gender, household income, educational level, and cognitive abilities. The sample is reduced (19 observations) due to missing values in control variables.

Among the control variables included in the regressions of columns (3) and (4), only the coefficients of the subject's age and educational level are statistically significant. In both studies, older subjects are more likely to reject unequal offers. For columns (3) and (4), respectively, the coefficient of age reports a *p*-value of 0.038 and 0.039 in Study 1 and of 0.052 and 0.059 in Study 2. The coefficients of educational level are negative and the *p*-values are 0.074 and 0.044, respectively, in Study 1 (in Study 2, educational level was constant across subjects as all were university students). These effects, although only marginally significant in some cases, can partially explain the higher proportion of subjects

setting their MAO to €10 in Study 1: subjects were on average older and less educated in that sample.

Table S2. Impact of DG behavior on the likelihood of rejecting unequal offers (Study 2)

	(1)	(2)	(3)	(4)
unfair – remaining	0.834**	0.835**	0.769*	0.788**
	(0.227)	(0.216)	(0.233)	(0.221)
p-value	0.000	0.000	0.001	0.000
fair – remaining	0.653**	0.650**	0.609**	0.612**
	(0.155)	(0.148)	(0.157)	(0.150)
p-value	0.000	0.000	0.000	0.000
unfair – fair	0.181	0.186	0.160	0.176
	(0.196)	(0.187)	(0.202)	(0.192)
p-value	0.356	0.321	0.428	0.360
order effects	YES	NO	YES	NO
pseudo R^2	0.0784	0.0412	0.0865	0.0473
log likelihood	-277.328	-288.522	-270.708	-282.314
LR (chi^2)	47.19*	24.80**	51.24*	28.03**
Controls	no	no	yes	yes
observations	623	623	615	615

Notes: Probit estimates. Standard errors in brackets. *, ** indicate significance at the 0.01 and 0.001 levels, respectively. In columns (3) and (4), controls are: age, gender, household income, and cognitive abilities (educational level is constant in this study). The sample is reduced (8 observations) due to missing values in control variables.

Note that, given that subjects made 5 decisions (summing up to 24 different combinations), in the regressions we control for all the possible orders of the decisions by adding a dummy variable for each order. However, a detailed analysis of order effects based on those 24 possible orders is extremely complex and probably meaningless. Thus,

we have focused on whether having decided first as dictator or as responder, irrespective of the order in which other decisions had been made, has an influence on the relationship we are investigating. We find that neither the binary variable "played dictator before responder" nor its interaction with any of the main explanatory variables (i.e., "unfair", "remaining" and "fair") yield significant estimates in any study (all ps>0.16; not reported).

Figure S3 plots the likelihood of subjects rejecting an unequal offer in the UG as a function of their transfers in the DG using a fractional polynomial model. The predictions of the model for Study 1 are displayed in panel a, and those for Study 2 in panel b. It can be observed that the above results are not due to the aggregation of subjects offering an amount between €0 and €10 within the group of "remaining" dictators. Specifically, Figure S3 shows that, in both studies, dictators offering €6 are the least likely to reject an unequal offer and that the likelihood increases as we move either to the left ("unfair" dictators) or to the right ("fair" dictators). A quadratic regression analysis (not reported) also reveals a significant U-shaped relationship (in all cases, both the linear and the quadratic term report p=0.000). Interestingly, in (S7) rejection behavior does not relate non-linearly (neither does linearly) to DG offers. This is possibly attributable to the fact that capturing such a non-linearity statistically requires a relatively large sample.

As mentioned earlier, the fact that subjects in Study 2 are less likely to set their MAO to €10 does not imply any change in terms of the relationship between such behavior and subjects' offers in the DG. This supports the existence of both spite- and fairness-driven punishers in the UG, even across very different samples.

Figure S3. Rejection of unequal splits as a function of DG offers. (a) Study 1, (b) Study 2

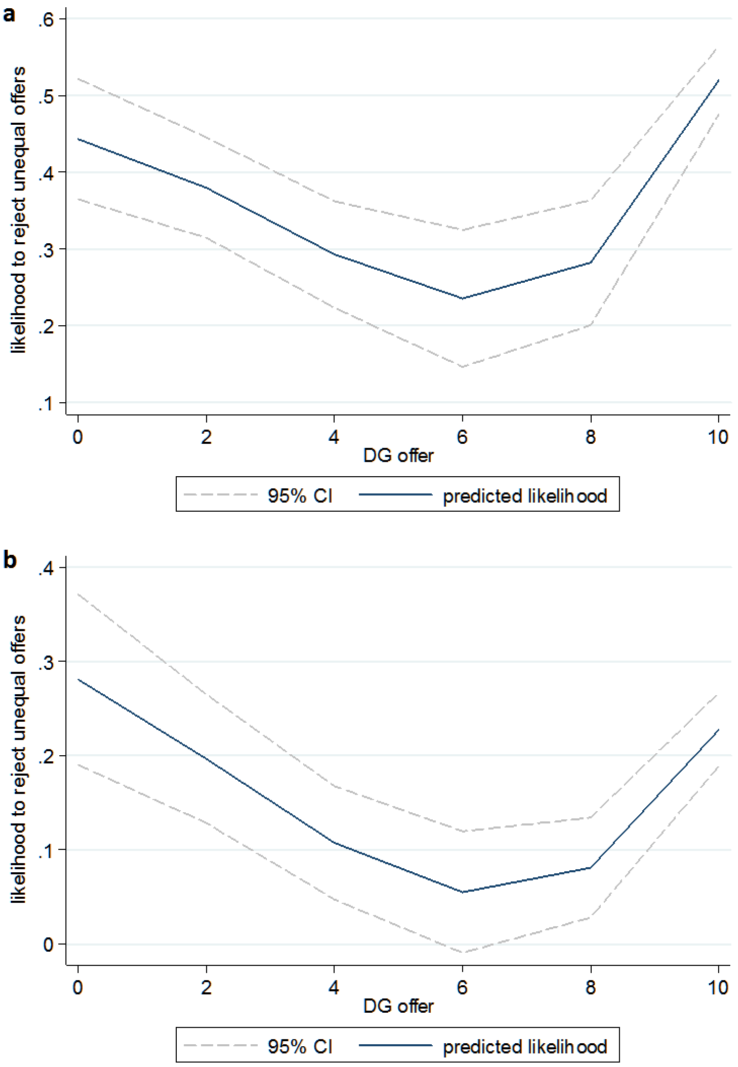

S3. Game instructions (Study 1)

In this part, you are going to make decisions with real money. This money comes from a national research project to be used specifically for this purpose. The money you will earn depends on 5 decisions that you are going to make later. Your decisions are totally independent of each other. You have to make the decisions that you prefer in each situation, without taking into account your decisions on the other situations. You are going to be paid for only one decision.

We will make a draw in which 1 out of 10 people will earn the real amount of money corresponding to the decision s/he has taken. Moreover, the decision that really "pays" among the 5 will be drawn randomly. For this reason, think carefully about your decisions because if you are drawn, what you have declared will be taken into account for your payment. In case you are drawn, we will make your payment within a few days.

The money you earn might also depend on the decisions of another person. Let us explain: for the 5 decisions you are going to be paired with another person. For each decision, your pair will be different and randomly selected. This person is another interviewee but none of you can identify the other, only that it is a person also living in Granada – we do not even know who s/he is. Anonymity is totally guaranteed. This is why not even we are going to know the decisions you make in this part. For this reason, I am going to give you a sheet to write down your answers. After you write down your answers, please place them in an envelope without letting us look at them. When I ask you, do not say your decisions out loud, just fill in the answer sheet.

Dictator Game:

For this decision, we give you 20€ in order for you to divide it between you and the other person. From this amount you can send the share you want to the other person, that is, you can send nothing, everything, or just a part. Obviously, the part that you do not send is for you to keep. How much money do you send to the other person? In the **BLUE** table you have to mark with a circle the number of euros you want to SEND to the other person. You can only choose even numbers: (0, 2, 4,…, 20).

Ultimatum Game (responder):

In this part, we give you 20€ in order for you to divide it between you and the other person. One of you is going to propose how to divide it, while the other can either accept or reject the proposed division. If s/he rejects it, none of the two will earn anything. For example: the one who decides the division sends 4€ to the other, keeping 16€ for him/herself and the other accepts it. Hence, the one who divides earns 16€ and the other,

who accepts the division, earns 4€. In contrast, if s/he does not accept the proposed division none of the two will earn anything. Understood? Decisions:

If you are the one who receives the money sent by the other person, you can accept or reject the division. If you accept the proposed division, you have to mark the **A** in the **YELLOW** table with a circle. If you reject the proposed division, mark the **R** but do not say out loud that you rejected it. If s/he sends you:

- 0€ and keeps 20€, you accept or reject the proposed division (A or R in the first cell of the YELLOW table). Remember that a rejection means that nobody earns anything.
- …
- 10€ and keeps 10€ (A or R in the last cell of the YELLOW table)

References

S1. Exadaktylos F, Espín AM, Brañas-Garza P (2013) Experimental subjects are not different. *Sci Rep* 3: 1213.

S2. Eckel CC, Grossman PJ (2000) Volunteers and pseudo-volunteers: the effect of recruitment method in dictator experiments. *Exper Econ* 3(2): 107-120.

S3. Forsythe R, Horowitz JL, Savin NE, Sefton M (1994) Fairness in simple bargaining experiments. *Games Econ Behav* 6(3): 347-369.

S4. Mitzkewitz M, Nagel R (1993) Experimental results on ultimatum games with incomplete information. *Int J Game Theory* 22(2): 171-198.

S5. Camerer CF (2003) *Behavioral Game Theory—Experiments in Strategic Interaction* (Princeton Univ. Press, Princeton).

S6. Henrich J et al. (2006) Costly punishment across human societies. *Science* 312(5781): 1767-1770.

S7. Yamagishi T et al. (2012) Rejection of unfair offers in the ultimatum game is no evidence of strong reciprocity. *Proc Natl Acad Sci USA* 109(50): 20364-20368.
